# Evaluation of CD8 T cell killing models with computer simulations of 2-photon imaging experiments

**DOI:** 10.1371/journal.pcbi.1008428

**Published:** 2020-12-28

**Authors:** Ananya Rastogi, Philippe A. Robert, Stephan Halle, Michael Meyer-Hermann

**Affiliations:** 1 Department of Systems Immunology and Braunschweig Integrated Centre of Systems Biology (BRICS), Helmholtz Centre for Infection Research, Braunschweig, Germany; 2 Institute of Immunology, Hannover Medical School, Hannover, Germany; 3 Institute for Biochemistry, Biotechnology and Bioinformatics, Technische Universität Braunschweig, Braunschweig, Germany; 4 Centre for Individualised Infection Medicine (CIIM), Hannover, Germany; 5 Cluster of Excellence RESIST (EXC 2155), Hannover Medical School, Hannover, Germany; National Institutes of Health, UNITED STATES

## Abstract

*In vivo* imaging of cytotoxic T lymphocyte (CTL) killing activity revealed that infected cells have a higher observed probability of dying after multiple contacts with CTLs. We developed a three-dimensional agent-based model to discriminate different hypotheses about how infected cells get killed based on quantitative 2-photon *in vivo* observations. We compared a constant CTL killing probability with mechanisms of signal integration in CTL or infected cells. The most likely scenario implied increased susceptibility of infected cells with increasing number of CTL contacts where the total number of contacts was a critical factor. However, when allowing *in silico* T cells to initiate new interactions with apoptotic target cells (zombie contacts), a contact history independent killing mechanism was also in agreement with experimental datasets. The comparison of observed datasets to simulation results, revealed limitations in interpreting 2-photon data, and provided readouts to distinguish CTL killing models.

## Author summary

Despite having a clear understanding of cytotoxic T lymphocyte (CTL) mediated cytotoxicity mechanisms, its quantitative dynamics remain unexplored at a cellular level. We developed an agent-based model to compare different hypotheses for mechanisms of CTL mediated cytotoxicity that could lead to an increased killing of infected cells at higher interactions with CTLs as seen *in vivo*. We showed that this behaviour can be explained by modulation of properties by infected cells or CTLs with increasing number of contacts. For the modulation, we compared two modes of signal integration and showed that the number of contacts is the main determinant of cell death. We also studied the impact of contacts between CTLs and apoptotic infected cells on observed killing properties.

## Introduction

Protection from intracellular pathogens like non-cytolytic viruses requires the detection and clearance of intracellularly infected cells. One of the most prominent cell-mediated protective immune effector mechanisms is the cytotoxic T lymphocyte (CTL) response. CTLs migrate within lymphoid tissues and can travel between infected organs and search for infected cells presenting foreign antigens. The motility properties, the capacity to find infected cells, and ultimately the decision to kill infected cells with minimal damage to healthy cells are critical factors for an optimal containment of infection.

Upon target cell encounter, CTLs can initiate contacts with infected cells that lead to the formation of an immunological synapse through which CTLs deliver perforin and granzymes to kill the infected cells [1,2]. Various mechanisms can be used by CTLs to kill infected cells, which include secretion of cytotoxic granules, surface-receptor mediated signalling, and secretion of cytokine [3]. Many of these protective functions of CTLs have been explored and are well understood [4]. However, until recently, most of these studies relied on *in vitro* CTL assays and on cell culture techniques. *In vitro* studies of killing dynamics at a cellular level have revealed that infected cells form simultaneous contacts with multiple CTLs [5].

To better quantify the results observed in experimental systems, analyses have been performed to calculate the per capita killing rate (PCKR) of CTLs and the death rate of infected cells. These include differential equation–based approaches with various spatial compartments described by their concentrations [6–9]. In a series of studies consisting of a simulated environment made to resemble a portion of the lymph node, Gadhamsetty et al. have shown that a double saturation function is the best fit for the killing rate [10,11]. This was done with a 2D cellular Potts model and was used to explore killing regimes such as monogamous killing, where one CTL can kill just one infected cell at a time; simultaneous killing, where one CTL can kill multiple infected cells at the same time; joint killing, where multiple CTLs kill a single infected cell; and mixed killing, where multiple CTLs can kill infected cells simultaneously.

To understand the dynamics of how CTLs interact and kill infected cells in a three-dimensional (3D) tissue *in vivo*, Graw et al. [12] developed a 3D model to look at CTL mediated killing through the lens of CTL motility. Indeed, the dimensionality of Potts models affects the efficiency of CTL mediated killing of target cells [13]. These models use an empirical probabilistic mechanism. Additionally, stochastic modelling approaches have shown that the size of the naïve T cell population affects the immune response and a bigger population size leads to a more efficient response [14]. However, the killing mechanisms at an individual cellular level remained unresolved.

Previously, we reported *in vivo* CTL-mediated killing kinetics analysed by 2-photon microscopy. We tracked CTLs interacting with virus-infected cells *in vivo* [15] inside lymph nodes with fluorescent reporter viruses that allow direct observation of the infected target cell over time. These studies relied on morphological disruption of the target cell as evidence for irreversible target cell death and showed that one CTL contact event typically did not suffice to achieve killing. Instead, the experimental data showed that target cells that were contacted multiple times by CTLs during the observation period were more frequently disrupted than target cells with one interaction. The observed probability of killing infected cells also showed an increase with increasing number of interactions with CTLs. This observation challenged the conventional view of CTL immunity, that only one contact suffices, and further suggests complex mechanisms by which CTLs adapt their killing efficiency or infected cells become more susceptible to death.

Here, we developed an agent-based model that reproduces the *in vivo* movement and interactions of CTLs with infected cells in 3D (see methods) and tested various hypotheses about which killing mechanisms at the cellular level can explain the quantitative killing dynamics observed in [15]. For each of the hypotheses, we also explored the impact of contacts between already apoptotic infected cells and CTLs, which we termed as “zombie contacts”. While earlier work using simpler models has shown that T cells stay in contact with their targets for longer time than the time of the lethal hit delivery [16–18], zombie contacts include the initiation of new contacts with apoptotic cells. The presence or the impact of zombie contacts on imaging data analysis remains unexplored.

The results from the model showed that retention of information about prior contacts, by either CTLs or infected cells or both, results in an increase of the observed probability of killing infected cells with more interactions. Strikingly, the presence of zombie contacts challenged the interpretation of CTL killing activity, because even when infected cells and CTLs do not modulate their properties, we saw an increase of the observed probability of killing infected cells with more interactions. With this model, we estimated the time taken by a cell to disappear once the decision for death has been taken and the PCKR. The model unravelled that different types of mechanisms exhibit different PCKR profiles suggesting that PCKR measurements could further pin down the mechanism at work.

## Results

### Agent-based model of T cell-mediated killing

**Fig 1:**
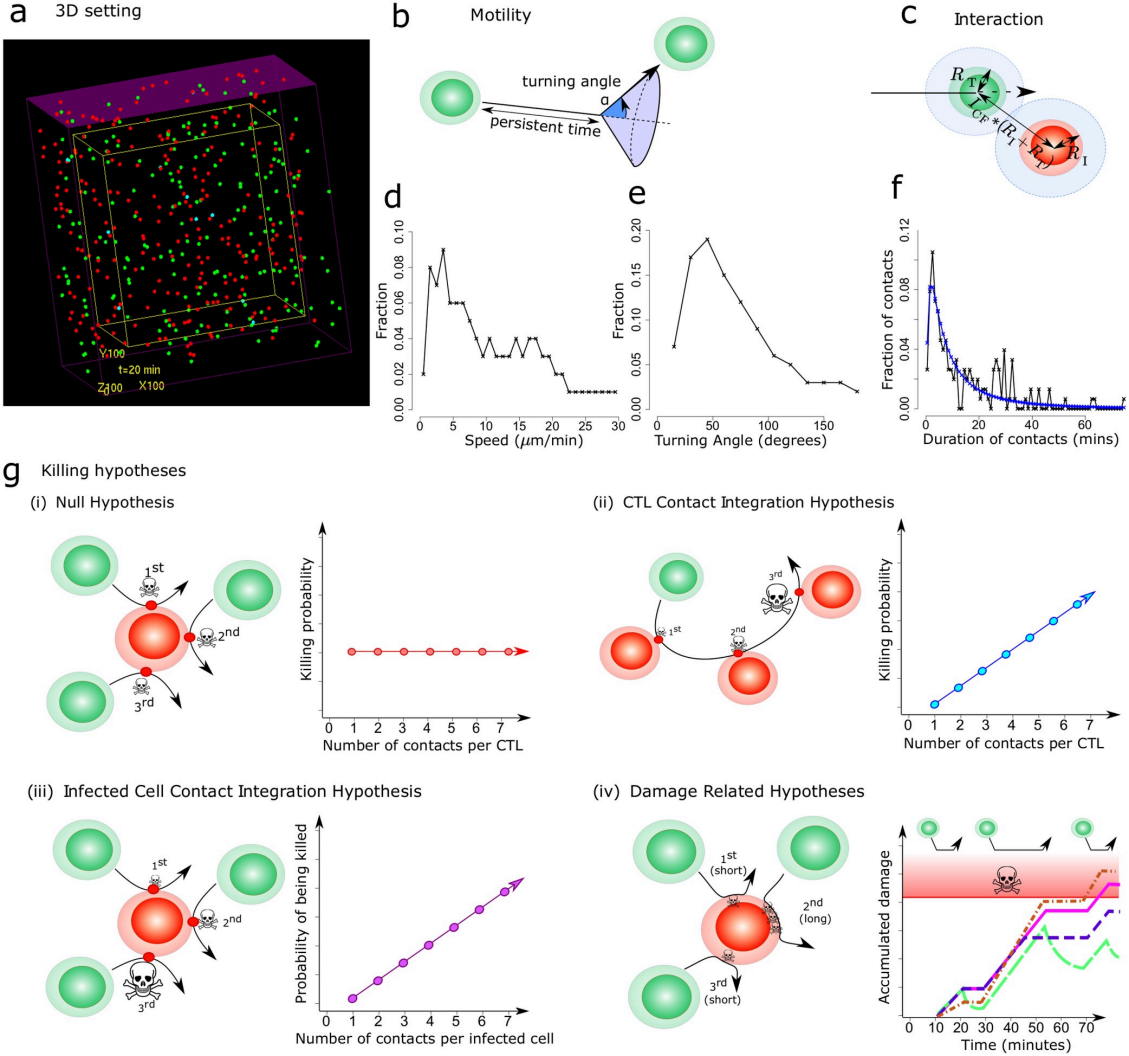
Design of the agent-based model for spatial CTL killing activity. (a) 3 dimensional view of the agent-based model, (b) CTLs in the model move in a straight direction until a persistence time is reached, then a new direction is randomly set according to an experimentally measured turning angle distribution, (c) a CTL interacts with an infected cell when they are in close proximity to each other, (d) binned speed distribution and, (e) binned turning angle distribution of CTLs obtained from experiments and used in the model, (f) binned distribution of contact durations between infected cells and CTLs (black- experimental data, blue- log normal plot fitted to experimental data and used in the model), (g) different killing hypothesis: (i) Null hypothesis: Infected cells and CTLs do not retain memory of prior contacts. Each contact is associated with a constant probability of death. The associated plot shows the behaviour of CTL killing properties with more interactions with infected cells; (ii) CTL contact integration: The CTLs retain memory of contacts and with each subsequent contact, CTLs become more lethal. The associated plot shows the behaviour of CTL killing properties with more interactions with infected cells; (iii) Infected cell contact integration: The infected cells retain memory of CTL contacts. The associated plot shows the behaviour of infected cell susceptibility to death with more interactions with CTLs; (iv) Damage hypotheses: The CTLs induce damage to the infected cells. The associated plot shows the behaviour of damage of infected cells with more interactions with CTLs for different hypotheses: damage (pink, solid line), damage and repair (green, long-dash line), saturated damage (purple, short-dash line) and CTL contact integration damage (orange, dot-dash line). The red area represents the damage greater than the threshold damage after which an infected cell dies.

In order to dissect the CTL-mediated killing activity observed *in vivo* in [15], we developed an agent-based model that reproduces the movement and interactions of CTLs with non-motile virus-infected cells in a three-dimensional context similar to the *in vivo* imaging setup [15] (Fig 1A) (see Methods). We modelled CTL migration to mimic T cell behaviour as observed by 2-photon microscopy (Fig 1B) and once the CTLs and the virus-infected cells are at a threshold distance from each other, an interaction is initiated (Fig 1C). The CTLs move in a direction and with a speed that we randomly picked from measured distributions (Fig 1D and 1E). The experimental speed and angle distributions are computed over time intervals of one persistent time, i.e. 2 minutes. Thus, after each persistent time, a new direction and speed are sampled from those distributions (see Methods). We focused on the case of CTLs reacting to antigen-expressing virus-infected cells with a relatively low average CTL speed [15]. The duration of an interaction is directly taken from experimental data (Fig 1F). The resulting model quantitatively reproduces CTL migration in the case of antigen recognition by peptide-MHC class I presentation on virus-infected cells.

Using this basic model setup, we compared different hypotheses regarding possible CTL-mediated killing mechanisms (Fig 1G). The (i) *Null hypothesis*: defined as a scenario with a contact history-independent CTL-mediated target cell killing, i.e. CTLs kill with equal probability at each CTL-target cell contact irrespective of the duration of contact between CTL and target. The considered alternative hypotheses are: (ii) *CTL contact integration*: at each contact, CTLs kill with a linearly increasing probability as they have interacted with more infected cells; (iii) *infected cell contact integration*: increased probability of death at higher number of contacts with CTLs; and (iv) *damage*: damage accumulation of infected cells with each contact with CTLs, proportional to duration of contact, possibly combined with repair of the cell, which is the *damage and repair hypothesis*, or with a limited damage per contact due to T cell exhaustion, which is *saturated damage* or a more mechanistic view of CTL contact integration where CTLs impart modulate damage rate with more CTL-target cell interactions, which is *CTL contact integration damage hypothesis*. The datasets used to discriminate between killing hypotheses are taken from [15] (S1 Fig).

### A contact history independent killing mechanism is in contradiction to 2-photon experiments

**Fig 2:**
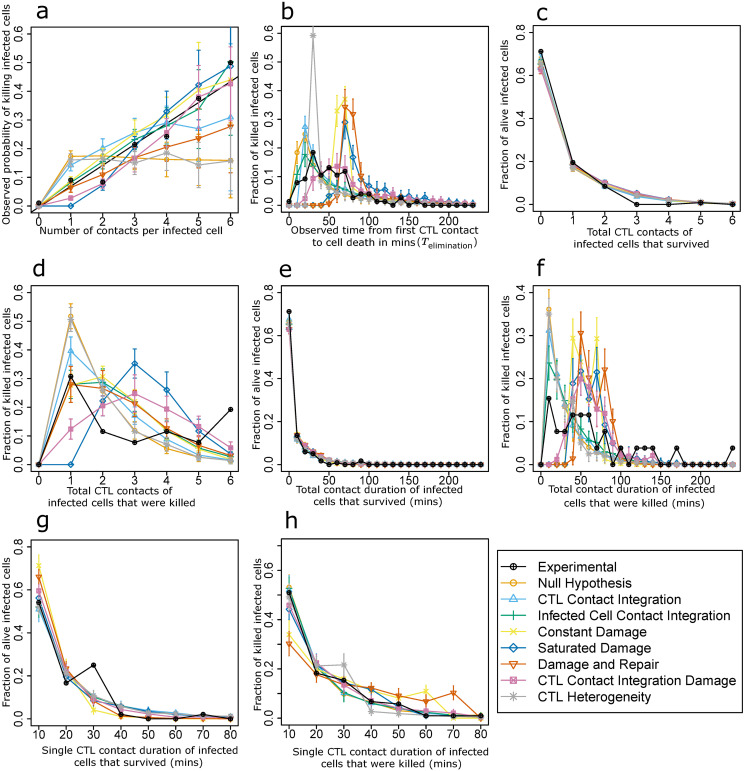
Analysis of in silico killing simulations in the absence of contacts between CTLs and apoptotic target cells (zombie contacts). Simulation results are compared with experimental measurements for different killing hypotheses in the absence of zombie contacts, using the best identified parameters for each hypothesis (S2 Fig). (a) Observed probability of killing infected cells in dependence on the number of interactions with CTLs, (b) distribution of observed times between first contact to a CTL and actual cell death for all killed infected cells, (c) distribution of the number of contacts with CTLs for all infected cells that survived during the observation period and, (d) were killed during the observation period, (e-h) distribution of total (e, f) and single (g, h) contact durations with CTLs for infected cells that survived during the observation period (e, g) and were killed during the observation period (f, h). Error bars represent SD from 30 simulations.

First, we investigated whether the observed properties of CTL-mediated killing seen in quantitative 2-photon imaging (see Supplement, S1 Fig) could be explained by a contact history independent killing mechanism (Null hypothesis). Although most of the model’s parameters are directly known from experimental measurements, the probability of a CTL to kill an infected cell and the time between activation of apoptosis and visual cell dissolution remained to be estimated. We identified the parameter values best reflecting the experimental data by minimizing the cost (Supplement S2A Fig, Methods). Only a restricted range of parameters were identified as giving rise to a low cost. The curves for each hypothesis using their best parameter set are shown in (Fig 2). For certain hypotheses, there were no parameter values that gave rise to an agreement between model results and experimental observation.

As expected in this model scenario (see Fig 2A–2H), the observed probability of killing infected cells (see Methods) remained constant with increasing number of interactions (Fig 2A), which is in contradiction to the experimental results (black lines). Thus, testing the Null hypothesis serves as a sanity check that the model works properly, and that the observed *in silico* probability of killing infected cells with more interactions reflects the cellular CTL-mediated killing mechanism in this context. Further, it shows that a simple contact history independent killing probability cannot explain the *in vivo* datasets of Halle et al. with this model setting [15].

As a variation of the contact history independent mechanism, we also investigated a CTL heterogeneity hypothesis where each CTL has an associated killing probability that remains constant irrespective of the number of prior contacts. This hypothesis too did not perform well when tested against the experimental datasets (see Fig 2A–2H). We further investigated the possibility of an infected cell heterogeneity hypothesis but it did not perform well either (see S9 Fig, see Methods).

### Contact integration mechanisms on infected cells or CTLs are compatible with 2-photon experiments

In order to explain the increase in observed probability of killing infected cells with each interaction with CTLs (Fig 2A, black line), we hypothesized that this could reflect priming of CTLs by modulation of CTL killing capacity each time they encounter an infected cell (Fig 1G (ii)). In this hypothesis, CTLs kill at each contact with a linearly increasing probability proportional to the number of previous target cell contacts of this CTL. The cost (quality) of simulations with different parameter values is shown, and an optimal parameter set could be identified (see Supplement, S2B Fig).

For this optimal parameter set, the observed probability of killing infected cells in dependence on the number of interactions with CTLs and the corresponding analyses for the other experimental datasets (Fig 2A–2H) showed a good agreement with the experimental results. Thus, the hypothesis that CTLs modulate their killing capacity is compatible with the experimental observations. As a sanity check for CTL contact integration hypothesis, we also compared the observed killing probability of infected cells for naïve and primed CTLs and observed that primed CTLs show a higher observed killing probability of infected cells (see Supplement, S4 Fig).

Next, we explored whether infected cells might become more sensitive to cytolysis with increasing numbers of CTL contacts (infected cell integration hypothesis, Fig 1G (iii)). The death susceptibility of infected target cells was modelled as a linearly increasing probability of death that is proportional to the number of prior CTL visits (see methods and Supplement, S2C Fig). The simulation with the best parameter set (Fig 2A–2H) was also in agreement with the experimental data suggesting that infected cells may get more susceptible to cell death with increasing number of CTL contacts.

There are certain qualitative mismatches between the experimental and model datasets but as we were unable to draw concrete conclusions on the basis of these mismatches, we relied on AIC values to describe the performance of the hypotheses.

### Damage accumulation of infected cells at a linear rate is not compatible with 2-photon experiments

Having established that infected cells retaining memory of prior cell contacts is a possible process, we wanted to elucidate the mechanisms by which infected cells could retain memory of previous CTL contacts. We hypothesized that infected cells get damaged by interacting CTLs and once the damage of an infected cell reaches a threshold value of 1 (100%), the cell dies. The three linear damage-based hypotheses that were considered were constant damage, saturated damage, and damage and repair (Fig 1G (iv)). While all other hypotheses have one parameter to describe the killing dynamics of infected cells by CTLs, saturated damage hypothesis and damage and repair hypothesis have two parameters.

The constant damage hypothesis assumes a constant rate of damage during each interaction. In the saturated damage hypothesis, the damage process by the CTL stops after the interaction exceeds a threshold time (*T*_max_). This hypothesis was proposed because CTLs have a storage of cytolytic granules and are possibly not able to sustain a damage process for very long interactions, reaching up to 40 minutes in the *in vivo* dataset. Therefore, a possible biological factor that affects the killing observations could be T cell exhaustion during single contacts. We assumed that the time lapse in between two contacts is long enough to allow the CTLs to recuperate and damage the next contacted infected cells again. In the damage and repair hypothesis, there is no T cell exhaustion. The CTLs damage the infected cells throughout the duration of the interaction but the infected cells also repair themselves.

The increase in observed probability of killing infected cells with increasing number of interactions with CTLs was recapitulated for the constant and the saturated damage hypothesis (Fig 2A) with the best parameter set in S2D and S2E Fig. However, the simulated observed time between the first contact to a CTL and the actual cell death (*T*_elimination_) did not coincide with the experimental distribution (Fig 2B).

The best damage rate was approximately *d* = 0.03 per minute for the constant and the saturated damage hypotheses. In order to achieve a damage level of 1, the total contact time required would be in the range of 30 minutes (see Methods) which is the minimum value for *T*_elimination_. This is a lower bound because we assumed that the time between the fate decision for death and the actual dissolution of the cell is negligible. For the saturated damage hypothesis, the value would exceed these 30 minutes (see Methods). For the damage and repair hypothesis, the optimal value for the damage rate was 0.03 damage per minute and the value for the repair rate was 0.009 per minute (S2F Fig). We did not find experimental data for repair kinetics pertaining to CTLs but repair after bacterial toxin induced pores has been estimated to take place over the scale of a few minutes [19]. We chose to try a large range of possible values for the repair rate including the scale of the repair rate of bacterial toxin induced pores. However, we did not find a good agreement of experimental and model results for any of the repair rates combined with any damage rate. The duration of one contact sufficient to induce a total damage of 1 was approximately 40 minutes (see Methods, Eq (5)). For an infected cell that died, *T*_elimination_ will also include the time that lapsed between consecutive contacts. As shown by the calculations above, for all of the damage-based hypothesis, no infected cell can have a *T*_elimination_ value less than 30 minutes which is in disagreement with the experimental data (Fig 2B). Thus, we conclude that none of the tested damage-based hypotheses are compatible with the experimental datasets.

### CTL contact integration damage could be a mechanism for contact memory retention by CTLs and infected cells

With the CTL contact integration and infected cell contact integration hypotheses, we have established that cells modulate their properties with cell contacts. The damage-based hypotheses were proposed to explore potential mechanisms through which infected cells retain memory of contacts. None of the infected cell damage hypotheses were in good agreement with experimental data. Thus, we proposed an additional damage-based hypothesis where the CTLs retain memory of prior contacts and have a higher damage rate at the end of each interaction. As a consequence, this hypothesis represents a situation where CTLs and infected cells modulate their properties with increasing number of contacts while infected cells accumulate damage from previous visits.

We identified the best parameter set (S2G Fig). Simulations for this hypothesis showed a good agreement with experimental data and also provided us with a potential mechanism for CTL and infected cell memory retention (Fig 2A–2H).

In contrast to linear damage, saturated damage, and damage and repair hypothesis, the damage rate for each CTL increases with increasing contacts with infected cells. As a consequence, infected cells see some CTLs with a higher damage rate early on. This circumvents the delay observed in the other damage-based hypotheses and reduces the time for observed time between the first contact to a CTL and the actual cell (*T*_elimination_) (Fig 2B). Thus, we are able to obtain a good fit between model and experimental data.

### Constant killing probability with the assumption of zombie contacts is compatible with 2-photon experiments

Virus-specific CTLs may interact with target cells even during the process of target cell death. Thus, a target might receive multiple CTL contact events, even though the fate decision to die has already been taken (“zombie contacts”). However, it was observed that once a target cell is disrupted, the remaining small remnants of the target cells are contacted by CTLs only sometimes [15]. We argued that most of these remnants are taken up by local dendritic cells and macrophages, thus preventing direct access of the CTLs to the membrane of the remnants.

It is unknown whether CTL interactions with target cells in the process of cytolysis might affect the analysis of CTL killing mechanisms and estimates of CTL killing efficiency. To address this question, we next tested whether the allowance of such zombie contacts impacts the observed probability of killing infected cells with increasing number of interactions with CTLs.

**Fig 3:**
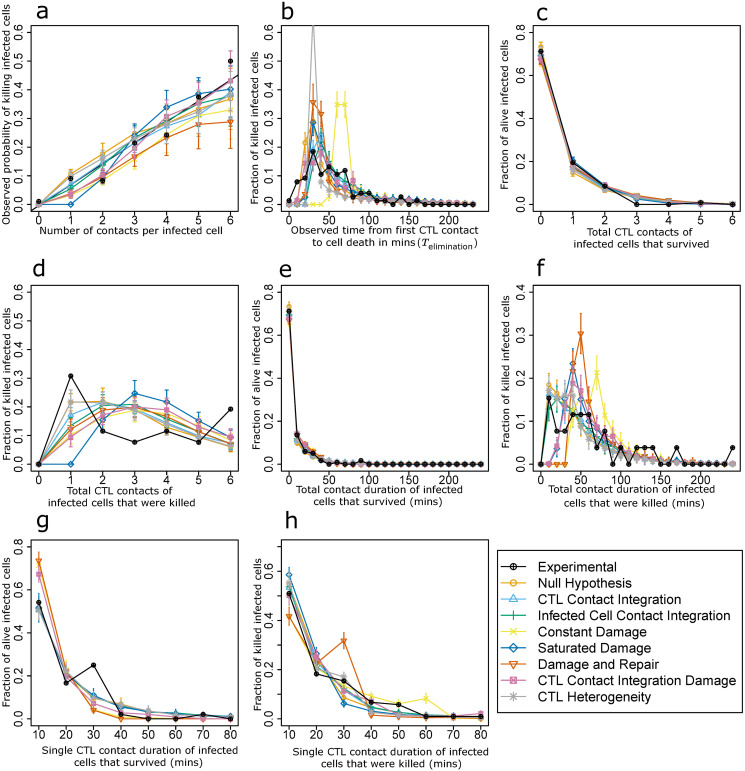
Analysis of in silico killing simulations in the presence of contacts between CTLs and apoptotic target cells (zombie contacts). Simulation results compared with experimental measurements for different killing hypotheses in the presence of zombie contacts, using the best identified parameters for each hypothesis (S3 Fig). Curves are depicted similar to Fig 2. Error bars represent SD from 30 simulations.

Simulations with the Null hypothesis with zombie contacts were performed with different killing probabilities and times for infected cell death. An optimal parameter set could be identified (see Supplement, S3A Fig). The simulations (Fig 3A–3H) show that, although CTLs mechanistically kill with equal probability at each contact in the model, in the presence of zombie contacts the observed probability of killing infected cells exhibits an increase with increasing number of interactions with CTLs (Fig 3A).

Due to zombie contacts, when following CTL killing activity *in vivo*, the time during which cells are already dying enters the analysis of the number of contacts. This leads to an overestimation of the number of contacts required for the decision of cell death. Therefore, even with a constant killing rate, cells with less contacts for cell death will be underestimated and cells with more contacts for cell death will be overestimated. This makes the observed increase in probability of infected cell death for the Null hypothesis in the presence of zombie contacts an artefact that looks similar to experimental results showing the significant impact of zombie contacts on the interpretation of the data. Hence, we concluded that the increase in observed probability of killing infected cells with each CTL interaction can be directly influenced by zombie contacts, even when the CTLs and infected cells do not adapt their properties. Interestingly, the Null hypothesis in presence of zombie contacts was compatible with all other datasets (Fig 3B–3H). In addition, after the 6^th^ contact, the simulations showed a saturation in the observed probability of killing infected cells while the experimental dataset continues increasing. Eventually, a longer observation time would increase the total number of observed contacts per target cells and allow further supporting or discarding the Null hypothesis as possible mechanism.

### Zombie contacts have a major impact on the interpretation of 2-photon experiments and on model selection

**Fig 4:**
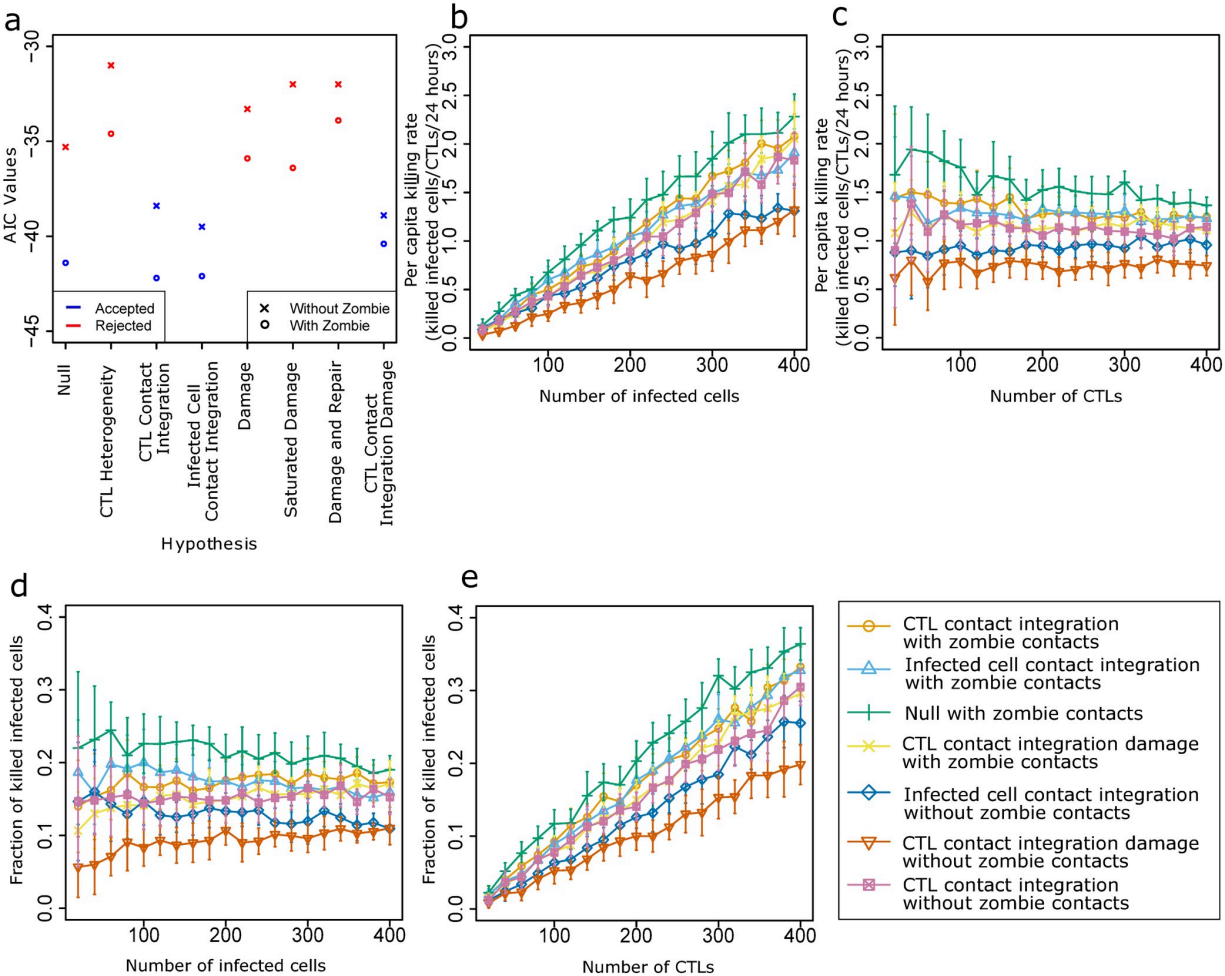
Model selection and parameter prediction. (a) Comparison of AIC values for all hypotheses corresponding to their respective lowest cost. The data points in blue show the hypotheses that gave a good agreement with experimental results. The data points in orange show the hypotheses that were rejected. (b, c) PCKR values for variable number of infected cells (b) and of CTLs (c), (d, e) Fraction of killed cells for variable number of infected cells (d) and of CTLs (e). Error bars represent SD from 30 simulations.

Considering the observed relevance of zombie contacts in the Null hypothesis, we revisited all other hypotheses to test whether addition of zombie contacts to our model impacts model performance in other scenarios as well (S3B–S3G Fig). CTL contact integration, infected cell contact integration, and CTL contact integration damage hypothesis gave results in agreement with the experimental results; but none of the linear damage-based hypotheses or the CTL heterogeneity hypothesis gave rise to plots that were in agreement with the experimental results. This implies that apart from the Null hypothesis, the ranking of the model hypotheses remained the same irrespective of the presence or absence of zombie contacts. However, all hypotheses showed a lower cost and a lower Akaike information criterion (AIC) value in presence of zombie contacts (Fig 4A, Tables 1 and 2).

**Table 1:** Lowest cost for all hypotheses in absence of zombie contacts in ascending order of AIC.

Hypothesis	Killing parameter	*T*_death_	Lowest cost	AIC
**Infected cell contact integration**	0.09	15 ± 2.5	4.35 × 10^−3^	−39.5
**CTL contact integration damage**	0.008	10 ± 2.5	4.70 × 10^−3^	−38.9
**CTL contact integration**	0.1	20 ± 2.5	4.99 × 10^−3^	−38.4
**Null hypothesis**	0.2	15 ± 2.5	7.35 × 10^−3^	-35.3
**Constant damage**	0.03	35 ± 2.5	9.37 × 10^−3^	-33.3
**Damage and repair**	*d* = 0.03, *r* = 0.009	40 ± 2.5	8.60 × 10^−3^	-32.0
**Saturated damage**	*d* = 0.03, *T*_*max*_ = 20	35 ± 2.5	8.64 × 10^−3^	-32.0
**CTL heterogeneity**	*k*_*m*_ = 0.18, *k*_*s*_ = 0.05	30 ± 2.5	9.80 × 10^−3^	-31.0
**Infected cell heterogeneity**	*d*_*m*_ = 0.1, *d*_*s*_ = 0.05	15 ± 2.5	1.29 × 10^−2^	-28.8

**Table 2:** Lowest cost for all hypotheses in presence of zombie contacts in ascending order of AIC.

Hypothesis	Killing parameter	*T*_death_	Lowest cost	AIC
**CTL contact integration**	0.14	35 ± 2.5	3.09 × 10^−3^	−42.2
**Infected cell contact integration**	0.18	35 ± 2.5	3.12 × 10^−3^	−42.1
**Null hypothesis**	0.35	25 ± 2.5	3.41 × 10^−3^	−41.4
**CTL contact integration damage**	0.014	10 ± 2.5	3.90 × 10^−3^	−40.4
**Saturated damage**	*d* = 0.06, *T*_*max*_ = 10	15 ± 2.5	5.01 × 10^−3^	-36.4
**Constant damage**	0.03	35 ± 2.5	6.82 × 10^−3^	-35.9
**CTL heterogeneity**	*k*_*m*_ = 0.3, *k*_*s*_ = 0.05	30 ± 2.5	6.27 × 10^−3^	-34.6
**Damage and repair**	*d* = 0.03, *r* = 0.009	5 ± 2.5	6.83 × 10^−3^	-33.9
**Infected cell heterogeneity**	*d*_*m*_ = 0.3, *d*_*s*_ = 0.05	15 ± 2.5	7.23 × 10^−3^	-33.4

### The per-capita killing rate and the fraction of killed cells could discriminate hypotheses

By selecting the optimal parameter and plotting the readouts obtained from the experiments, various hypotheses were discarded. The seven hypotheses that give a good agreement with all datasets (Fig 4A, blue dots) in ascending order of cost are: (i) CTL contact integration hypothesis in presence of zombie contacts, (ii) Infected cell contact integration in presence of zombie contacts, (iii) Null hypothesis in presence of zombie contacts, (iv) CTL contact integration damage hypothesis in presence of zombie contacts, (v) infected cell contact integration in absence of zombie contacts, and (vi) CTL contact integration damage hypothesis in absence of zombie contacts, and (vii) CTL contact integration hypothesis in absence of zombie contacts.

We sought to predict properties of the different hypotheses that could further help to discriminate them or to design new predictive experiments. One property is the observed PCKR of CTLs, as a measure of 3D population killing efficiency. The PCKR is defined as the number of infected cells killed per CTL in 24 hours. Another important measure is the fraction of killed infected cells defined as the ratio of killed infected cells to the total number of infected cells in the system.

To understand how the number of infected cells affects the PCKR values, the number of CTLs was kept constant at 200 while the infected cell number was varied from 20 to 400 (Fig 4B). The other parameter values were kept fixed at the optimal parameter set obtained from the respective cost heatmaps of each hypothesis. The increase of PCKR values was highest for the Null hypothesis but for all seven hypotheses, the values of PCKR increased with increasing number of infected cells.

Next, to elucidate the effect of the number of CTLs on the PCKR, the number of infected cells was kept constant at 250 while the CTL numbers were varied from 20 to 400 (Fig 4C). Surprisingly, even when the ratio of infected cells to CTLs is high, the values of PCKR remained constant for all hypotheses except the Null hypothesis for which we saw a small drop in values of PCKR with increasing number of CTLs.

In the same spirit, we investigated the fraction of infected cells that are killed by CTLs for different initial numbers of infected cells. A surprising result is that even for a low number of infected cells, CTLs fail in killing all infected cells within the simulated 4-hours (Fig 4D).

In contrast to the results obtained for variable number of infected cells, the fraction of killed infected cells increased with number of CTLs for all hypotheses (Fig 4E). At higher numbers of CTLs, a much larger fraction of infected cells is killed. Taken together, these results suggest that by keeping all other factors of a system constant and varying either the number of infected cells or the number of CTLs, we can differentiate between different killing hypotheses by analysis of PCKR and fraction of killed cells.

In order to further understand how CTL and infected cell density impact the behaviour of the system, we also investigated the extent of CTL coverage for varying numbers of infected cells by plotting the fraction of infected cells with no contacts with CTLs (S5 Fig). The fraction of cells which get no contact with CTLs remained constant even at higher numbers of infected cells. This is consistent with the observation that the fraction of killed infected cells remained constant with varying numbers of infected cells (Fig 4D).

## Discussion

Following up on *in vivo* 2-photon microscopy-based imaging of CTL-mediated immunity [15], we developed a 3-dimensional agent-based model to visualize and quantify the dynamics of virus-infected cells and CTLs. Different hypotheses on how CTLs kill infected cells were compared to observed CTL killing dynamics. Cells other than infected cells that might be able to interact with CTLs by processing and presenting extracellular antigens with MHC class I molecules to them were not included. Already with this restricted model complexity, a combination of measured datasets was required to discriminate different hypotheses.

To discriminate between the hypotheses, we evaluated a cost between simulations and all combined datasets. While the experimental data for observed probability of killing infected cells included data for up to 14 contacts with CTLs, the experimental data points beyond the 6^th^ contact only represent a small number of cells. Due to this, only the 6 first interactions are included in the cost calculation. Thus, experiments with more data points at higher CTL contact numbers could help in model selection.

For all hypotheses, a better fit with experimental data was found in the presence of zombie contacts, suggesting that there is a substantial time between the decision of death and actual disappearance of the cell, and that it is critical to include this parameter. Importantly, there is no reliable *in vivo* reporter system for this hypothetical mechanism available. Notably, caspase activity reporter systems might be useful [20], but it is typically not clear at what stage of caspase activity the pro-cell death pathway passes an “irreversibility threshold” in the context of an ongoing CTL attack. Thus, better *in vivo* sensors of target cell viability will be helpful to better define the “point of no return” of CTL-mediated target cell killing.

The results have been summarised for each hypothesis below:

- Null hypothesis: In the absence of zombie contacts, the hypothesis was rejected as it expectedly did not give rise to an increase in observed probability of killing infected cells with increasing CTL contacts. On the other hand, on including the effect of zombie contacts, the hypothesis shows a good agreement of model results with experimental results due to the existence of contacts that take place after the cell has taken the decision to die. Therefore, dying cells have an overestimation in the number of CTL contacts they received.
- CTL contact integration hypothesis and infected cell contact integration: Both the contact integration hypotheses, with or without zombie contacts, showed a good agreement of experimental and model datasets indicating that memory retention of prior contacts by cells is a potential mechanism of CTL mediated killing.
- Linear damage, damage and repair, and saturated damage hypotheses: None of the damage-based hypotheses provided a good agreement of model output with experimental datasets. The low damage rate needed to give rise to the observed probability of killing infected cells took too long to kill infected cells and led to a much larger *T*_elimination_ value compared to the data.
- CTL contact integration damage hypothesis: The CTL contact integration hypothesis combined memory retention and damage hypotheses and provided a good agreement of model result in the absence and presence of zombie contacts. This is expected because it is an extension of CTL contact integration, and shows that damage accumulation in this context is compatible with the data, contrary to the previously explored damage hypotheses. This is because the damage rate associated with each CTL increases with increasing contacts, which circumvents the longer *T*_elimination_ time observed in the damage-based hypotheses by faster killing.
- CTL heterogeneity hypothesis: The CTL heterogeneity hypothesis was rejected as we were unable to reproduce the datasets for increase in observed probability of killing infected cells and *T*_elimination_. Without zombie contacts, this is expected as the probability to die at next contact is not correlated with the interaction history. With zombie contacts, although an increase in killing per contact is observed, as in the null hypothesis, the *T*_elimination_ times are not well recapitulated. This is because the probability of CTLs with a higher probability of killing eliminate most infected cells with just one contact. Thus, it generated a too high peak for *T*_elimination_. This is structural incompatibility between quick killing and longer *T*_elimination_ values.

We showed that retention of information about prior contacts, by either CTLs or infected cells or both, is compatible with all the observed datasets. Surprisingly, in the presence of contacts between dying infected cells and CTLs (zombie contacts) a contact history independent killing mode (Null hypothesis) gave rise to an increase in the observed probability of killing with each contact of the infected cell with a CTL. This raises the question if in the experimental system, the killing is really getting more efficient or is an artefact of zombie contacts. Thus, the hypothetical possibility of zombie contacts introduces another uncertainty into the analysis of live-imaging data. To further enhance our understanding of this system, it is imperative to be able to distinguish zombie contacts from other contacts in an experimental setup. In the case that zombie contacts actually happen, the present simulations might be used to reconstruct the real killing efficiency given the measured data. We have shown the interpretation of 2 photon imaging data is affected by the existence of zombie contacts. The conclusions obtained in the presence and absence of zombie contacts are different. One can argue that the contacts between CTLs and target cells, or parts of target cells during cell death, is something that very likely does exists. However, the importance of such interactions is unknown, in particular, the length of such interactions and the point at which the infected cell initiates death is unknown. A system with smaller *T*_death_ value would behave in the same way as if there are no zombie contacts. Therefore, further exploration of different hypotheses was imperative to investigate potential killing mechanisms that would perform well in the absence of zombie contacts.

In the simulations, we sampled the duration of contacts from just one distribution. This implicitly assumes that CTL contacts with zombie and alive cells are of equal duration. The possibility of two distinct distribution of duration of contacts could be tested through aforementioned sensors to detect apoptotic phases of infected cells. Inclusion of different distributions of contact durations may further progress our understanding of the system.

We established that a positive modulation of CTL killing with each contact with infected cells is a viable hypothesis. However, CTLs in the context of chronic infections or cancer can get exhausted [21,22]. Exhaustion is usually defined as a reduced protective response in the face of ongoing antigenic activation. E.g. during virus infections exhausted CTLs lose activities like direct killing and cytokine production [23,24]. These mechanisms are in general viewed as negative modulation of the killing capacity of CTLs [25]. Thus, on the single cell level, a CTL that interacted with a sequence of multiple target cells would rapidly become less cytotoxic, being able to kill fewer-and-fewer target cells. Interestingly, none of the parameter values tested supported such a decreased killing efficacy of infected cells per contact. This suggests that, at the time point studied and within the duration of analysis of 240 minutes, exhaustion upon multiple contacts is not dominant. Indeed, this is in agreement with studies that show the time scale over which CTL exhaustion is observed is in the range of days or even weeks [26]. Thus, an observation period that lasts 90 to 240 minutes will not show CTL exhaustion. For CTLs, in our settings, the average time lapse between consecutive contacts is ~17 minutes which is comparable to the average duration of contacts of ~12 minutes implying that cells spend most of their time in contacts and it would be hard to observe the phenomenon of CTL inactivation. In real life, the CTLs lose their activation state, and with longer / longitudinal experimental datasets, it could become possible to include this mechanism.

On the contrary, the simulation suggests that CTLs get more lethal with increasing numbers of contact. The CTL contact integration hypothesis naturally gives rise to a diverse population of CTLs that show heterogeneity during killing. This is in agreement with studies showing that CTLs have dissimilar killing properties that contribute to a robust T cell response [27,28].

Interestingly, a high variation was observed in the ability of individual CTL to impart calcium flux on infected cells [15] suggesting functional heterogeneity amongst CTL killing capacity. Due to the complex patterns of calcium signaling, and difficulty in linking calcium to a functional decision of cell death, we could not directly relate the experimental percent of calcium flux and the fraction of interactions triggering death in our model. In all of the hypotheses, we have considered, we have already integrated some degrees of variability associated at each interaction between the infected cells and CTLs. For instance, the killing in all hypotheses has been described as a probability, indicating that most interactions are not successful at killing. Further, for the damage-related hypotheses, the duration of each contact creates a different type of variability. To explore the consequence of dissimilar CTL killing properties, we explored a CTL heterogeneity hypothesis (see Methods) and the hypothesis was rejected due to a large AIC value).

Signal integration from multiple CTLs on the side of infected cells was also found in agreement with the experimental data. We, thus, proposed damage-based hypotheses as possible mechanisms by which the retention of contacts by infected cells is implemented. While the infected cell contact integration hypothesis describes a system where the number of CTL contacts determines cell death, the damage-based hypotheses also resolve the duration of the interactions, i.e. a longer interaction leads to a greater damage [29–31]. The simulation results suggest that for CTLs, the number of contacts with infected cells rather than the time integration of signals are critical for CTL killing properties. The model we have described in our study consists of simple hypotheses for killing infected cells and by using the experimental datasets, we have managed to discriminate between hypotheses. However, we are trying to reproduce complex mechanisms in real biological systems by using simple hypotheses which cannot completely reproduce the experimental observations. Additionally, the experimental data would consist of noise and comparing them to simple mechanisms for infected cell killing gives rise to qualitative mismatches between experimental and model results. These qualitative mismatches have not been used to accept or reject any hypotheses. Instead, we have relied on AIC values as an objective measure to determine the performance of killing hypotheses.

An additional complexity that arises in experimental systems is that at the time of imaging, there is no way of knowing the contact history of each CTL or infected cell and the start of the movie is not the true start of the infection. Thus, it is unclear whether *T*_elimination_ < 30 minutes really reflects killing in less than 30 minutes, or whether the infected cell or CTL has already undergone several unseen interactions prior to imaging that would lead to enhanced killing or death probabilities. To investigate this phenomenon, we ran simulations but we ignored the first hour of the model history by starting the observation after 60 minutes such that the cells in the system would have a prior history and previous interactions that are not a part of the observation. The AIC values for the hypotheses (S1 and S2 Tables) showed that the general order of the hypotheses which performed best remained the same.

The 2-photon imaging datasets (S1 Fig), despite encompassing multiple levels of information on duration of contacts and tracking of cell contacts, were not sufficient to identify whether the CTL-mediated killing rate is constant or integrated by the CTL or infected cell or both. However, we here showed that more indicators could be used to discriminate these remaining hypotheses. A first indicator is the killing efficiency of CTLs, as defined by PCKR of CTLs [32]. The PCKR values obtained for the final hypotheses are different for varying numbers of initial CTLs and infected cells. However, the differences are not large and in real biological systems there may be noise due to which PCKR might not be a value that can be accurately estimated. This suggests that while a careful measurement of PCKR values with well-defined numbers of CTLs and infected cells (Fig 4B and 4C) could be suitable to discriminate the hypotheses, currently the PCKR values from our model cannot provide any further insights.

In the model described in this study, we have not included the role that chemoattractants could play in CTL motion properties as the observations from [15] provided no evidence to support the role of chemokines in CTL localization in these particular experiments. Nonetheless, the presence of chemotaxis in CTL motion has been observed in other studies [33] and the model can be extended to include the role of chemoattractants as seen in infected tissue provided new datasets are generated to monitor CTL motility with respect to infected cells positions for instance [34]. The directed motion and subsequent recruitment of CTLs as a consequence of chemotaxis could give rise to an increase in number of times an infected cell is contacted and subsequently, an increase in elimination of infected cells. Modelling this would give an insight into whether faster CTL recruitment would significantly impact on infected cell death.

A recent study [35] reported a higher PCKR value at low CTL to infected cell ratio. In the simulations, we observe a similar behaviour when we vary the number of infected cells while keeping the number of CTLs constant (Fig 4B). But a higher PCKR value at low CTL to infected cell ratio was not in agreement with the results obtained when keeping the number of infected cells constant and varying the number of CTLs. Thus, our results suggest that instead of the ratio of CTLs to infected cells, the number of infected cells impact the PCKR values.

In [15], the PCKR values were found to range from 2 to 16. While PCKR is used extensively to study CTL killing properties, this range of values is not enough yet to reject or accept any hypotheses. The significance of cytotoxicity mediated by CTLs and the impact of the number of cells on PCKR and on the fraction of killed infected cells are especially important due to the role of T cells in viral infections such as HIV, viral pneumonia and other diseases such as cancer [36,37].

At very low numbers of simulated infected cells, the CTLs still fail to kill all infected cells which contradicts the rapid CTL mediated killing seen in other studies [38]. This could be a consequence of the assumed random cell movement and raises the question whether directed cell movement where CTLs actively migrate towards infected cells [34,39] is important to ensure successful elimination of more infected cells.

The fraction of total killed infected cells shows a linear increase with increasing number of CTLs, implying that CTL mediated killing could follow mass action killing kinetics. Previous studies have shown that T cells show mass action killing kinetics in the spleen [40] and our results suggest similar dynamics in the lymph node. Interestingly, our findings show that CTL concentration determines the efficiency with which infected cells are killed. This is in agreement with prior studies which showed that it is not the infected cell to CTL ratio but the concentration of the CTL that had the dominant influence on the efficiency of killing [41].

A limitation of the estimated parameters obtained from the agent-based model is that they fit best for a specific system that describes an *in vivo* setup. As established with the PCKR and fraction of killed infected cell analyses, the results differ based on initial number of infected cells and CTLs. Thus, the parameters identified in the paper hold true for a certain experimental set-up and cannot be taken for different initial conditions. The PCKR values calculated in this study are specific for rapid CTL killing observed in the presence of a modified murine cytomegalovirus (MCMV). The type of target cell might strongly affect CTL killing rates and mechanisms. So far, we have studied CTL-mediated killing of MCMV-infected stromal cells and MVA-infected macrophages in the mouse lymph node [15] but these values are likely different under varying killing rates. For example, hematopoietic cells with high surface peptide-MHCI levels might be an easy target for CTLs [42]. In contrast, killing in the tumor microenvironment has been observed to be a much slower process and take place over the course of hours [43]. Thus, a direct *in vivo* comparison of CTL killing rates would be very informative. Nonetheless, the benefit of the model is the ease with which it can be adapted to various other initial conditions. Consequently, while the agent-based system described in the paper is designed for a particular *in vivo* system, it can be applied to different setting by varying the parameters and can be used to study the killing mechanisms under different conditions. Other than to study killing mechanisms, it may be used to study cell activation that is based on integrating signals from cell-cell-interactions.

With the existing datasets and observations, we were unable to differentiate between infected cell contact integration and CTL contact integration hypotheses using an agent-based *in silico* approach. We also explored a system with low number of CTLs and high number of infected cells and vice versa (see S6 and S7 Figs). In this case, as one type of cell is a limiting factor, we can see how the system behaves at extreme limits. However, even with these initial conditions, we are unable to identify the suitable hypothesis as all the hypotheses do not show a significantly different behavior from each other. It is conceivable that the actual behaviour of the system could be best defined by a mixed hypothesis which includes some aspects of different hypotheses. We tested one such hypothesis where the first contact has a higher likelihood of killing a target than subsequent contacts (see S8 Fig). However, even with an added layer of complexity, the hypothesis did not perform better than the simple hypothesis discussed in the paper.

There might be multiple mechanisms at work that might contribute to a certain “memory” effect in the interactions between CTLs and target cells. On the target cell side, accumulation of mitochondrial damage might be possible [44]. Once a certain threshold of damage is reached, mitochondrial outer membrane potential destabilization could be a central regulation event. Also, caspase cascades offer ample opportunity for signal integration during the CTL-mediated induction of target cell death [3]. So far, the exact number, amount and timing of these signals have not been recorded *in vivo*. Thus, we are not able to implement this level of detail in the current simulations. On the CTL side, multiple signalling pathways might be able to generate memory effects: T cell receptor stimulation leads to many parallel effects on the T cells. Some of these downstream effects could e.g. increase cytokine production and release, increase production of cytotoxic granule content, increase helper cytokine or chemokine secretion upon subsequent target cell encounters [45]. We also propose studying the killing of infected cells by CTLs in real time by measuring the cleavage of fluorogenic caspase substrates [46]. The obtained observations would help in identifying the lethal contact and showing whether zombie contacts exist or not. With this information, we could then discriminate between relevant hypotheses from our study bringing us a step closer to understanding mechanisms of CTL mediated cytotoxicity.

## STAR methods

### Experimental dataset

We used the experimental dataset published by Halle et al. [15] to investigate which killing hypothesis can best explain the quantitative properties of CTL killing activity *in vivo*. Briefly, the experimental setup consists of mice infected with a modified reporter virus. We have used both murine cytomegalovirus (MCMV-3D-delta-vRAP) and modified vaccinia virus Ankara (MVA-OVA-mCherry) that both do not inhibit MHC class I peptide presentation, that both express a fluorescent protein (mCherry), and that further express a specific OVA protein-derived peptide. In parallel, we used CD8 T cells specific for the OVA-derived peptide (OT1 mouse model) that express another fluorescent protein that were adoptively transferred to the mice (prior to T cell priming and later infection with the reporter viruses). In that setting, virus-infected cells are red fluorescent while green GFP^+^ CTLs specifically recognize infected cells. Two-photon microscopy was used to observe micro-anatomical regions containing virus-infected cells inside lymph nodes. In these experiments, only a small portion of the lymph node tissue is monitored. CTLs are recruited to interact with the infected cells in this region. The movement of the T cells is tracked, together with the duration and numbers of contacts between T cells and infected cells, and finally the time at which infected cells disappear. Here, we described an agent-based model designed to reproduce this experimental setting.

### Three-dimensional setting and movement of cells

The CTLs and infected cells are positioned in a continuous three-dimensional space of 700×700×700 *μ*m (Fig 1A). The space is assumed to be periodic along x- and y-axis such that if a cell leaves from one side, it re-enters from the opposite end keeping the other coordinates same and keeping the same velocity vector. Along the z-axis the borders are impermeable, i.e. cells cannot leave from the top of the tissue. While the cells can leave through the lower boundary along z-axis in the experiments, in our simulations, we assumed a closed system along the lower and upper z-boundary. CTLs are placed randomly in the whole space whereas the infected cells are placed only in the top 40% (*Z*_lim_) of the space with respect to the z-axis, as seen *in vivo* [15] to reflect the microscopy depth of nearly 200 to 300 μm. The remaining space is used as pool of CTLs which can migrate to the site of infection.

Regarding modelling of CTL migration, we assumed a core repulsion of the nucleus that is assumed to be 50% of the overall size of the cell. Cells are initially positioned in the space such that no two nuclei physically overlap with each other. The CTLs have a radius of 4.8 *μ*m (*R*_T_) and the infected cells have a radius of 5.1 *μ*m (*R*_I_) (directly taken from experimental observations from [15]).

CTLs are allowed to move and infected cells are stationary. The CTLs carry a current direction of movement and a speed value (velocity). The initial speed value is chosen from the distribution from (Fig 1D), and the initial directions are assigned randomly. The CTLs have a constant persistence time of 2 minutes which is the time that a particular cell moves in one direction before changing directions [47]. When the persistence time of a CTL is reached, it is assigned a new direction of movement and a new speed taken from the distributions obtained from experimental data. Initially, the time that each cell has been moving in the same direction is assigned as a random value between 0 and the persistence time to avoid synchronization. The model of migration is similar to what has been described by Beauchemin et al. [48] but in contrast, we did not observe a momentary pause in T cell motion when they changed their direction of motion at the end of each persistence time.

### Collision detection and interaction between cells

At each time step of 0.1 minutes, the following tasks are carried out:

(a) **Collision**: CTLs that are currently not interacting with an infected cell are checked for movement:

If the CTL has been moving in the same direction for equal or more than the persistent time, the CTL is assigned a new speed (Fig 1D). The direction of the CTL is determined by choosing a turning angle value from the experimentally observed distribution which was computed over the same time period as implemented in the model (Fig 1E). The new direction in which the CTL moves is calculated based on this value. The plane in which this turning angle is implemented is chosen randomly. The experimental distribution of the turning angles was computed by measuring the values at intervals of 2 minutes which is the value chosen for the persistence time in our ABM model. The order of updating the movement of the CTLs is executed in an unsynchronized manner to avoid any bias.

Then, the CTLs are checked for collisions. The CTLs are moved in their direction of movement as far as possible within the distance they should move during that time-step, until they reach another cell, according to nucleus hard-core repulsion. More precisely, a cell is reached when the distance between the centre of the CTL being moved and the other cell is *C*_CF_(*R*_T_ + *R*_I_) in the case of when the other cell is an infected cell and is *C*_CF_(*R*_T_ + *R*_T_) when the other cell is a CTL. Here *R*_T_ and *R*_I_ are the radii of the CTL and infected cells, respectively, and *C*_CF_ defines the fraction of the cell radius taken by the nucleus. If a collision takes places, the CTL changes its direction and resumes motion in another direction.

(b) **Interaction initiated**: The cells are checked for interaction. Each CTL can interact with just one infected cell at a given time but an infected cell can have multiple interactions simultaneously. Multiple simultaneous encounters are considered to have an additive effect and we assume that there are no cooperative effects at this scale. If the distance between a free infected cell and a CTL is less than *I*_CF_(*R*_T_ + *R*_I_), where *I*_CF_ is the interaction confinement factor, interaction is initiated and the cell stops moving for the entire interaction duration. The factor for *I*_CF_ is set as 1.5 to account for the fact that cells may extend pseudopodia to interact with another cell. Note that an interaction can be initiated before cells collide into each other.

(c) **Interaction terminated**: Once an interaction has been initiated, the duration of that particular interaction is decided based on a predefined distribution. The experimental data for duration of interaction has very few points and to achieve a better sampling, the distribution of interaction duration is chosen from a fitted log normal distribution, leading to a mean of 2.1 and a standard deviation of 1.2 (Fig 1F).

When the duration of the ongoing interaction between a CTL and an infected cell exceeds the interaction time assigned in the beginning, the interaction is broken off and the CTL is assigned a new speed in a direction chosen randomly. Further interactions are not allowed for the CTL for a time period as long as the persistence time after an interaction is terminated.

(d) **Hypotheses for target cell death**: Infected cells that are interacting with CTLs are checked for death. Target cells for which the cell death decision has been initiated do not vanish from the system immediately but persist for a certain time to die called *T*_death_. The value of *T*_death_ is treated as an unknown parameter and is taken to be a constant value. The interaction is terminated when either the value for *T*_death_ for the infected cell is reached or when the interaction time is completed, whichever happens first. The death decision is based on the following possible hypotheses:

1. Null hypothesis: The probability of infected cell death remains constant with increasing number of CTL contacts. The cell death decision is taken at the end of each interaction.

2. Infected cell contact integration: The probability of target cell death increases linearly with increasing number of CTL contacts. At each contact, the probability of cell death is given by *k*_I_ * *C*_I_ where *k*_I_ is constant and *C*_I_ is the number of contacts that the infected cell has had including the current one. Similar to the Null hypothesis, the cell death decision is taken once at the end of each interaction.

3. CTL contact integration: the killing capacity of the T cell is modulated with increasing number of contacts. For the case of positive modulation, at each contact, the probability of cell death is given by *k*_T_ * *C*_T_ where *k*_T_ is constant and *C*_T_ is the number of contacts that the CTL has had. Here, the CTL gets more lethal with increasing number of contacts with infected cells. On the other hand, a negative modulation of CTL killing capacity is given by *k*_T_/*C*_T_ where an increasing number of contacts with infected cells leads to a long-term exhaustion of the T cells. Death decision is taken at the end of each interaction.

4. CTL heterogeneity hypothesis: To account for the variation in CTL killing properties, we included a hypothesis where all CTLs have an individual associated killing probability sampled from a normal distribution of mean, *k*_m_ and standard deviation, *k*_S_.

5. Infected cell heterogeneity hypothesis: Further, to account for the variation in infected properties, we included a hypothesis where all infected cells have an individual associated probability of getting killed sampled from a normal distribution of mean, *d*_m_ and standard deviation, *d*_S_.

6. Damage: As a possible detailed mechanism by which infected cells get more susceptible following CTL attack, infected cells accumulate damage. All infected cells have an initial damage value of 0. The damage is assumed to be proportional to the duration of contact and is computed at each time step during every interaction for an infected cell according to Eq (1):

$$\begin{array}{c} \frac{dI}{dt}=n_{CTL}(t).d \end{array}$$ (1)

where *I* is the damage of infected cell, *d* is the damage rate and *n*_CTL_(*t*) is number of CTLs that are interacting with the infected cell at time *t*. From Eq (1), it can be seen that for an infected cell to reach a damage of 1, a total contact with CTLs of 1/*d* minutes is required. For an infected cell, this is the minimum duration of contact after which it initiates apoptosis. Once an infected cell reaches a damage of 1, the death process is initiated.

7. Saturated damage: All infected cells have an initial damage of 0. Damage of infected cells during a single interaction with CTLs is limited to a certain duration *T*_max_. For longer interactions, the CTLs cease to damage the infected cells. For each CTL, the damage induced in a particular infected cell during interaction is given by:

$$\begin{array}{c} \frac{dI}{dt}=\sum_{i=1}^{i_{T}}\{\begin{array}{c} d,if\;T_{i}<T_{max}\\ 0,if\;T_{i}\geq T_{max} \end{array} \end{array}$$ (2)

where *I* is the damage of the infected cell, *d* is the damage rate, *T*_*i*_ is the time duration of the *i*^th^ interaction and *i*_T_ is the total number of CTL contacts that the infected cell is currently having.

Similar to the damage hypothesis outlined above, an infected cell needs an interaction of at least 1/*d* minutes to acquire a damage of 1. But if the value of 1/*d* exceeds *T*_max_, the infected cell will accumulate the damage over a series of contacts. Thus, the time to reach a damage of 1 would also include the time between two consecutive contacts and would be greater than 1/*d*.

8. Damage and repair: In addition to damage by CTLs, infected cells can also repair themselves. The repair rate is constant. At each time step time, the damage for an infected cell is updated according to:

$$\begin{array}{c} \frac{dI}{dt}=n_{CTL}(t).d-rI \end{array}$$ (3)

where *I* is the current damage of the infected cell, *d* is the damage rate, *n*_CTL_(*t*) is the number of CTLs interacting with the infected cell at time *t* and *r* is the repair rate.

The damage evolution during the first contact follows Eq (4):

$$\begin{array}{c} I=\frac{d}{r}(1-e^{-rt}) \end{array}$$ (4)

Then, the duration of the first contact to reach a damage of 1 (*t*_complete_) is given by:

$$\begin{array}{c} t_{complete}=\frac{-1}{r}\text{ln}\left(1-\frac{r}{d}\right) \end{array}$$ (5)

Contacts between CTLs and already dying cells, i.e. a target cells during the *T*_death_, are named “zombie contacts”. In the simulations, zombie contacts can be allowed or not, in which case CTLs ignore the dying cells as soon as the cell death decision has been established for that target cell. If zombie contacts are allowed, they do not contribute to the cell death decision but are nonetheless observed as contacts when analysing the data.

After *T*_death_ elapses, the dying cell vanishes from the simulation.

9. CTL contact integration damage hypothesis: the damage inflicted by each T cell is modulated with increasing number of contacts. At each contact, the damage inflicted is given by:

$$\frac{dI}{dt}=k_{T}(t).C_{T}$$

where *k*_T_ is constant and *C*_T_ is the number of contacts that the CTL has had. Thus, the CTLs get more lethal while the infected cells retain memory of previous contacts by damage accumulation. This hypothesis serves as a combination of an increase in infected cell susceptibility and an increase in CTL lethality. By implementing this hypothesis, as a damage-based hypothesis, we are introducing only one unknown parameter. In contrast, implementing a combination of CTL contact integration and infected cell contact integration would lead to two or more unknown parameters.

### Finding best parameter sets

To compare the model results with the experimental results, we compared the following 8 datasets (see S1 Fig, S2 Data):

1. Observed probability of killing infected cells with a particular number of interactions with CTLs (S1A Fig). In the model, the number of infected cells that died at exactly the *i*^th^ interaction is normalized by the total number of infected cells with at least *i* interactions.
2. Distribution of the observed time between first CTL contact and the observed time of cell disruption (*T*_elimination_) (S1B Fig). In the model, this distribution is obtained by monitoring all cells from their first contact to a CTL, counting the number of killed cells in time bins, and normalising these with the total number of killed cells.
3. Distribution of the number of contacts with CTLs for infected cells that died during the observation period (S1C Fig). In the model, the number of CTL contacts is saved with each cell and a histogram is generated at the end of the simulation and normalized with the total number of dead cells at the end of the simulation.
4. Distribution of the number of contacts with CTLs for infected cells that survived the observation period (S1C Fig). In the model, same procedure as in point 3 is used but for cells alive at the end of the simulation.
5. Total duration of contact with CTLs for infected cells that died during the observation period (S1D Fig). In the model, each cell in contact to CTL increases a clock and a histogram on time bins is generated at the end of the simulation and normalized by the total number of dead cells at the end of the simulation.
6. Total duration of contact with CTLs for infected cells that survived the observation period (S1D Fig). In the model, same procedure as in point 5 but for cells alive at the end of the simulation.
7. Distribution of single CTL contact durations for infected cells that died during the observation period (S1E Fig). In the model, at each contact with CTLs a clock is running and the time when the cells detach is saved. For all dead cells, these times are recollected in a histogram on time bins at the end of the simulation and normalized with the total number of contacts of all dead cells.
8. Distribution of single CTL contacts duration for infected cells that survived the observation period (S1E Fig). In the model, same procedure as in point 7 but for cells alive at the end of the simulation.

Although most parameters are directly taken from data (Table 3), each hypothesis carries its set of unknown parameters, like a killing probability or damage rate, and *T*_death_. For each parameter set, we ran 30 simulations and calculated the respective cost for each of the 8 datasets described above and for each dataset the average cost was calculated over these 30 simulations. This gave us 8 values which were the average costs for each of the 8 datasets described above. When comparing different parameter sets, the formula in Eq (6) is used to compute the cost for each of the 8 datasets described above:

$$\begin{array}{c} C_{x}=\frac{1}{n}\sum_{1}^{n}\left[\sum_{i=1}^{N_{x}}{\left(E_{x,i}-M_{x,i}\right)}^{2}\right] \end{array}$$ (6)

where *C*_x_ is the average cost of the *x*^th^ dataset, *n* is the total number of simulations run for each parameter set, *N*_x_ is the total number of data points of the *x*^th^ experimental dataset, *E*_x,*i*_ is the *i*^th^ data point of the *x*^th^ experimental dataset and *M*_x,*i*_ is the *i*^th^ data point of the *x*^th^ model dataset with this parameter set. The subscript *x* represents the 8 datasets described above.

**Table 3:** Parameters fixed from data.

Parameter	Value
Number of CTLs (*N*_T_)	200
Number of infected cells (*N*_I_)	250
Percent of z-axis occupied by infected cells (*Z*_lim_)	40%
Radius of CTLs (*R*_T_)	4.8 *μ*m
Radius of infected cells (*R*_I_)	5.1 *μ*m
Interaction confinement factor (*I*_CF_)	1.5
Collision confinement factor (*C*_CF_)	0.5
Persistent time (*T*_pers_)	2 minutes
X-axis dimension of space (*X*_dim_)	700 *μ*m
Y-axis dimension of space (*Y*_dim_)	700 *μ*m
Z-axis dimension of space (*Z*_dim_)	700 *μ*m
Simulation (*T*_Sim_)	240 minutes
Speed	Taken from distribution (Fig 1D)
Turning angle	Taken from distribution (Fig 1E)
Interaction duration	Taken from distribution (Fig 1F)

The 8 separate costs were averaged to compute the mean cost for a particular parameter set using equation:

$$\begin{array}{c} C=\frac{1}{e}\sum_{x=1}^{e}C_{x} \end{array}$$ (7)

where *C* is the average cost for a particular simulation set, *e* is the total number of datasets (e = 8 in the current study).

For each hypothesis, the average costs are plotted in a heatmap comparing different combination of parameter values. Then, the parameter set with the lowest cost for each hypothesis was used to run the simulation to get the results. For the first dataset, since the experimental data points beyond the 6^th^ contact only represent a small number of cells, only the 6 first interactions are included in the cost calculation.

Details of AIC values are shown in (S1 Data). We also computed the Kolmogorov-Smirnov test to compare experimental and model datasets for all hypotheses (provided in Supplementary data as Excel files, S3 and S4 Data) but to distinguish between hypotheses, we used the AIC values.

## Supporting information

S1 Fig Experimental readouts used to compare experimental data with simulation data. (a) Fraction of killed infected cells at each interaction. The number of contacts are serial contacts and do not necessarily indicate contacts taking place simultaneously, (b) Distribution of observed times between the first contact to a CTL and the actual cell death for all infected cells that were killed. These measurements also include the time period that lapses between consecutive contacts, (c) Distribution of the number of contacts with CTLs for all killed and alive infected cells during the observation period. (d, e) Distribution of total (d) and single (e) contact durations with CTLs for killed and alive infected cells during the observation period.
(EPS)

S2 Fig Heatmaps for all hypotheses in absence of zombie contacts. (a) Null hypothesis; (b) CTL contact integration hypothesis; (c) Infected cell contact integration hypothesis; (d) Constant damage hypothesis; (e) Saturated damage hypothesis; (f) Concomitant damage and repair hypothesis; (g) CTL contact integration damage hypothesis. Each point on the heatmap is obtained by calculating the average cost over 30 simulations for the respective parameter combination. ‘X’ represents the parameter combination with the lowest cost. For saturated damage hypothesis and damage and repair hypothesis, there are three variable parameters and the lowest costs were found scanning the 3D parameter space.
(EPS)

S3 Fig Heatmaps for all hypotheses in presence of zombie contacts. (a) Null hypothesis; (b) CTL contact integration hypothesis; (c) Infected cell contact integration hypothesis; (d) Constant damage hypothesis; (e) Saturated damage hypothesis; (f) Concomitant damage and repair hypothesis; (g) CTL contact integration damage hypothesis. The heatmaps are obtained using the same conditions described in S2 Fig.
(EPS)

S4 Fig Observed probability of killing infected cells for naïve and primed CTLs. To compare the behaviour of primed and naive CTLs, the CTL contact integration in the presence of zombie contacts for the optimal parameter set was executed. The simulations were run for 480 minutes. While 500 infected cells were present in the system initially, 250 of them are not visible to the CTLs for the first 240 minutes. These infected cells become visible to CTLs by turning on their antigen expression at the end of 240 minutes. Thus, these infected cells interact with primed CTLs that have become more efficient as a consequence of prior interactions with infected cells. The plots show the observed probability of killing infected cells for the 250 infected cells present from time 0 to 240 minutes (red) and for the 250 infected cells for which the antigen expression was turned on from 240 to 480 minutes (orange). The observed probability of killing infected cells was much lower for naive CTLs and indicates that primed CTLs did become more efficient at eliminating infected cells.
(EPS)

S5 Fig Fraction of infected cells which did not get contacts with CTLs for varying numbers of infected cells. (EPS)

S6 Fig Analysis of in silico killing simulations for a CTL population half the population size described and studied in the model. (a) Observed probability of killing infected cells in dependence on the number of interactions with CTLs, (b) distribution of observed times between first contact to a CTL and actual cell death for all killed infected cells, (c) distribution of the number of contacts with CTLs for all infected cells that survived during the observation period and, (d) were killed during the observation period, (e-h) distribution of total (e, f) and single (g, h) contact durations with CTLs for infected cells that survived during the observation period (e, g) and were killed during the observation period (f, h). Error bars represent SD from 30 simulations.
(EPS)

S7 Fig Analysis of in silico killing simulations for an infected cell population half the population size described and studied in the model. Curves are depicted similar to S6 Fig. Error bars represent SD from 30 simulations.
(EPS)

S8 Fig Analysis of in silico killing simulations for infected cell contact integration hypothesis where the first contact has a higher likelihood of killing a target than subsequent contacts. Curves are depicted similar to S6 Fig. Error bars represent SD from 30 simulations. The AIC for the hypothesis in absence of zombie contacts is -37.1 and in the presence of zombie contacts is -39.7. Both these values fall in the middle of the range of AIC values calculated for all hypotheses as shown in Tables 1 and 2.
(EPS)

S9 Fig Analysis of in silico killing simulations for infected cell heterogeneity hypothesis. Simulation results are compared with experimental measurements for the infected heterogeneity hypothesis using the best identified parameters. (a) Observed probability of killing infected cells in dependence on the number of interactions with CTLs, (b) distribution of observed times between first contact to a CTL and actual cell death for all killed infected cells, (c) distribution of the number of contacts with CTLs for all infected cells that survived during the observation period and, (d) were killed during the observation period, (e-h) distribution of total (e, f) and single (g, h) contact durations with CTLs for infected cells that survived during the observation period (e, g) and were killed during the observation period (f, h). Error bars represent SD from 30 simulations.
(EPS)

S1 Table Lowest cost for all hypotheses with unknown history in absence of zombie contacts in ascending order of AIC. (DOCX)

S2 Table Lowest cost for all hypotheses with unknown history in presence of zombie contacts in ascending order of AIC. (DOCX)

S1 Data AIC values normalized by standard deviation for all hypotheses. (XLSX)

S2 Data Supplementary information text. (DOCX)

S3 Data Kolmogorov-Smirnov test for all hypotheses in the absence of zombie contacts. (XLSX)

S4 Data Kolmogorov-Smirnov test for all hypotheses in the presence of zombie contacts. (XLSX)
