# Supplementary material for: Evaluation of CD8 T cell killing models with computer simulations of 2-photon imaging experiments
Source: PLoS Comput Biol. 2020 Dec 28;16(12):e1008428. doi: 10.1371/journal.pcbi.1008428 (PMC7793284; doi:10.1371/journal.pcbi.1008428)
Supplement: S1 Table — (DOCX) [file pcbi.1008428.s010.docx]

*Table S1: Lowest cost for all hypotheses with unknown history in absence of zombie contacts in ascending order of AIC*

| **Hypothesis** | **Killing parameter** | $\boldsymbol{T}_{\mathbf{death}}$ | **Lowest cost** | **AIC** |
| --- | --- | --- | --- | --- |
| **CTL contact integration** | $0.1$ | $20\pm2.5$ | $8.89\times{10}^{-3}$ | $-33.8$ |
| **CTL contact integration damage** | $0.008$ | $10\pm2.5$ | $9.46\times{10}^{-3}$ | $-33.3$ |
| **Infected cell contact integration** | $0.09$ | $15\pm2.5$ | $1.05\times{10}^{-2}$ | $-32.7$ |
| **Constant damage** | $0.02$ | $10\pm2.5$ | $1.07\times{10}^{-2}$ | $-32.3$ |
| **Null hypothesis** | $0.2$ | $25\pm2.5$ | $1.37\times{10}^{-2}$ | $-30.6$ |
| **Saturated damage** | $d=0.02, T_{\max}=50$ | $10\pm2.5$ | $1.05\times{10}^{-2}$ | $-30.4$ |
| **Damage and repair** | $d=0.03, r=0.009$ | $10\pm2.5$ | $1.09\times{10}^{-2}$ | $-30.2$ |
