# Supplementary material for: Evaluation of CD8 T cell killing models with computer simulations of 2-photon imaging experiments
Source: PLoS Comput Biol. 2020 Dec 28;16(12):e1008428. doi: 10.1371/journal.pcbi.1008428 (PMC7793284; doi:10.1371/journal.pcbi.1008428)
Supplement: S2 Table — (DOCX) [file pcbi.1008428.s011.docx]

Table S2: Lowest cost for all hypotheses with unknown history in presence of zombie contacts in ascending order of AIC

| **Hypothesis** | **Killing parameter** | $\boldsymbol{T}_{\mathbf{death}}$ | **Lowest cost** | **AIC** |
| --- | --- | --- | --- | --- |
| **CTL contact integration** | $0.1$ | $30\pm2.5$ | $6.36\times{10}^{-3}$ | $-36.5$ |
| **Infected cell contact integration** | $0.12$ | $30\pm2.5$ | $8.31\times{10}^{-3}$ | $-34.3$ |
| **CTL contact integration damage** | $0.01$ | $15\pm2.5$ | $8.74\times{10}^{-3}$ | $-33.9$ |
| **Null hypothesis** | $0.3$ | $35\pm2.5$ | $8.79\times{10}^{-3}$ | $-33.9$ |
| **Constant damage** | $0.03$ | $45\pm2.5$ | $9.16\times{10}^{-3}$ | $-33.5$ |
| **Saturated damage** | $d=0.03, T_{\max}=45$ | $25\pm2.5$ | $9.06\times{10}^{-3}$ | $-31.6$ |
| **Damage and repair** | $d=0.07, r=0.056$ | $10\pm2.5$ | $9.11\times{10}^{-3}$ | $-31.6$ |
