# Supplementary material for: Evaluation of CD8 T cell killing models with computer simulations of 2-photon imaging experiments
Source: PLoS Comput Biol. 2020 Dec 28;16(12):e1008428. doi: 10.1371/journal.pcbi.1008428 (PMC7793284; doi:10.1371/journal.pcbi.1008428)
Supplement: S2 Data — (DOCX) [file pcbi.1008428.s013.docx]

**Supplementary Information**

*In vivo* time-lapse imaging of single CTLs during attack of virus-infected cells allows the detection of target cell death in relation to CTL behaviour. However, these data come with a couple of limitations. First, whether a CTL-target cell interaction is really “active”, meaning that during T cell-target cell contact a pro-death signal is transmitted, is impossible to detect. Even using calcium reporter expression in the virus-infected target cells, it was not possible to predict which CTL contact event leads to target cell death. In contrast, we observed a substantial heterogeneity in the CTL-target cell interactions. Many CTL contact events were not followed by a detectable calcium signal in the targets. Thus, the exact duration, function and “lethality” of a single CTL-target cell interaction is unknown. Another limitation of *in vivo* imaging is the restricted observation period: imaging can be done for maximum a couple of hours. Overall, it remains very difficult to record a time-series of images from a complex in vivo organ. Therefore, all available observations were pooled, with an average duration of the observation in the range of 1-3 hours. Notably, all CTL-target cell interactions before the start of the observation cannot be quantified. The experimental details with their limitations are discussed in detail in the paper by Halle et al [1].

The datasets discussed above share a complex correlation with each other that is difficult to extract from just the data points. For example, the data points plotted on the x-axis in Figs S1a and S1b are the number of contacts per infected cell and the observed time from first CTL contact to cell death ($T_{\mathrm{elimination}}$ ) , respectively. These two values are connected as infected cells with higher contacts with CTLs would have a higher $T_{\mathrm{elimination}}$. However, the correlation cannot be extracted as it depends on a number of stochastic factors such as distribution of time lapse between consecutive contacts and probability of a CTL contacting an infected cell. Therefore, no variables show a direct or linear correlation with each other. For this reason, all datasets are used separately to compute the cost and all contribute to the cost value equally.

**References:**

1. Halle S, Keyser KA, Stahl FR, Busche A, Marquardt A, Zheng X et al. In vivo killing capacity of cytotoxic T cells is limited and involves dynamic interactions and T cell cooperativity. Immunity. 2016 Feb 16;44(2):233-45.
